# Circulating miR-92a, miR-143 and miR-342 in Plasma are Novel Potential Biomarkers for Acute Myeloid Leukemia

**DOI:** 10.22088/acadpub.BUMS.6.2.2

**Published:** 2017-05-21

**Authors:** Amr Rafat Elhamamsy, Muhammad Suleiman El Sharkawy, Ahmed Farouk Zanaty, Mohammed Ahmed Mahrous, Ahmed Ezzat Mohamed, Eslam Ahmed Abushaaban

**Affiliations:** 1 *Department of Clinical Pharmacy, School of Pharmacy, Tanta University, 31516 Tanta, Egypt.*; 2 *Department of Clinical Pharmacy, Tanta Cancer Center, 31527 Tanta, Egypt.*

**Keywords:** Leukemia, myeloid, acute, diagnosis, microRNAs

## Abstract

MicroRNAs (miRNAs) are small non-coding RNAs that function as post-transcriptional gene expression regulators. The expression profiling of miRNAs has already entered into cancer clinics as diagnostic and prognostic biomarkers to assess tumor initiation, progression and response to treatment in cancer patients. Recent studies have opened the way for the use of circulating miRNAs as non-invasive diagnosis and prognosis of Acute myeloid leukemia (AML). The aim of this study was to identify plasma miR-92a, miR-143 and miR-342 expression signatures in AML patients to introduce new markers for establishing AML diagnosis and prognosis. Blood samples were collected from 65 AML patients and 50 controls. The expression of three target miRNAs (miR-92a, miR-143 and miR-342) was measured using quantitative real-time PCR method. Plasma levels of miR-92a, miR-143 and miR-342 were significantly lower in AML patients in comparison with control group. Receiver operator characteristic (ROC) analysis revealed that the sensitivity and specificity values of miR-92a were 81.5% and 94%, respectively, with a cut-off value of 0.704. The sensitivity and specificity values of miR-143 were 87.7% and 80%, respectively, with a cut-off value of 0.65. The sensitivity and specificity values of miR-342 were 75.4% and 90%, respectively, with a cut-off value of 0.479. Our findings suggest that plasma miR-92a, miR-143 and miR-342 could be promising novel circulating biomarkers in clinical detection of AML.

Acute myeloid leukemia (AML) is a malignant disorder of clonal hematopoietic stem cell, characterized by the rapid proliferation of leukemic blasts. The results of AML are the abnormal accumulation of immature cells and suppression of cells involved in normal hematopoiesis (1, 2). AML has a variable prognosis, which can range from few-weeks survival to complete remission and cure (3). The 5-year disease-free survival in adult AML patients is age-dependent with the poorest survival in patients older than 65 (4). Cytogenetic and molecular analyses are very important in predicting the remission and survival rates of AML patients according to the World Health Organization (WHO) categorization of AML (5). However, the prognosis of an AML patient is difficult to accurately estimate due to the diverse molecular mechanisms of AML development. Therefore, identification of novel biomarkers will lead to better treatment for AML and better prediction of chemotherapy response in these patients.

MicroRNAs (miRNAs) are a family of small non-coding RNA molecules with 18-25 nucleotides in length. MiRNAs function as a group of negative regulators of post-transcriptional gene expression by targeting the 3’-untranslated regions of the target mRNAs, resulting in the suppression or degradation of the target mRNAs (6). MiRNAs are involved in numerous physiological and biological functions such as proliferation, differentiation, organogenesis, embryogenesis, and apoptosis (7). Moreover, various diseases have shown aberrant expression and dysregulation of miRNAs, for example, cancer, lung, renal and hepatic diseases (8, 9). Dysregulation of miRNAs plays a major role in oncogenesis through the abnormal activation of oncogenes and the silencing of tumor suppressor genes. Therefore, several studies suggested the therapeutic targeting of specific miRNAs as a potential modality of suppressing tumors (10). Furthermore, expression profiles of miRNAs in plasma and serum have been used as non-invasive biomarkers to categorize specific types of cancers (11). Recent studies have highlighted the aberrant expression profiles of various miRNAs in different tissues including plasma in AML patients. Hence, plasma miRNAs represent novel biomarkers and valuable tools in diagnosing AML and estimating prognosis of AML patients (12, 13).

Several studies reported that miR-92a, miR-143 and miR-342 were dysregulated in different AML cell lines and tumor samples (14–17). Thus, studying the expression profile of these miRNAs in plasma presents new non-invasive biomarkers for this type of hematologic malignancy. In the present study, we examined alterations in the expression of miR-92a, miR-143 and miR-342 in plasma samples from AML patients and healthy volunteers to determine the significance of these miRNAs as diagnostic tools for AML. We also analyzed the correlation between the expression of these miRNAs and the clinicopathological features of AML.

## Materials and methods

**Patients and samples**

The present study was conducted on 65 plasma samples provided by AML patients, who were clinically diagnosed with AML and confirmed by bone marrow aspiration and biopsy at Tanta Cancer Center and the Department of Oncology, Faculty of Medicine, Tanta University, from May 2013 to September 2015. The diagnosis of AML was made according to a morphologic assessment of the Wright-Giemsa stained smears of the bone marrow aspirates along with special stains and immunophenotyping by flow cytometry. Laboratory investigation included conventional and molecular cytogenetic analyses. The AML patients (median age: 56 years; range: 43─68 years) were classified according to the French–American–British (FAB) classification systems (18). This study included AML patients with classification of M0 (2 cases), M1 (20 cases), M2 (26 cases), M4 (9 cases), M5 (7 cases), and M7 (one case).

In addition, 50 healthy volunteers, recruited by the Blood Transfusion Unit, Faculty of Medicine, Tanta University, were enrolled (median age: 55 years; range 42─67years) and served as the control group. Subjects with comorbidities and chronic diseases, e.g. cardiovascular disease, renal or liver disease were excluded from AML and control groups. Patients with metastasis or any other type of malignancy were excluded from both groups. No chemotherapy was administered prior to participation in the study. All participants were recruited and enrolled in the study after providing written informed consent for the use of their blood samples for research purposes and all study protocols were approved by the Ethics Committee of Faculty of Pharmacy, Tanta University. All clinical and pathological data were collected from medical records for the analysis of differences among groups. The clinical and pathological characteristics of the AML patients and normal controls are shown in Table 1. Blood (10 ml) was drawn on Ethylene diamine tetra acetic acid (EDTA) from an antecubital vein in AML patients or healthy control subjects using standard percutaneous venipuncture. Plasma was separated by centrifugation at 3000 rpm for 10 min at 4 °C, and then recentrifuged at 5000 rpm for 5 min to obtain cell-free plasma, which was stored at −80 C until use. Only non-hemolyzed plasma samples were used in our study, as haemolysis affects miRNA levels. Hemolysis was detected by spectral analysis at 541 nm (19, 20).

**Plasma miRNA assay**

Total RNA with preserved miRNAs was extracted from 200 µl plasma with the miRNeasy extraction kit (Cat No. 217184) (Qiagen, CA, USA) according to the manufacturer's instructions for isolation of total RNA, and eluted with 50 µl of RNase-free water. The isolated RNA was assessed and quantified by using Nano-Drop ND-1000 spectrophotometer (Thermo Fisher scientific, Waltham, USA) and the concentration of total RNA was about 5 ng/µl.

Total RNA was reversely transcribed using miScript II RT Kit (Cat No. 218161) (Qiagen, CA, USA) according to the manufacturer's instructions with 25 ng of total RNA per reaction.

The expression of miR-92a, miR-143 and miR-342 was evaluated by real-time polymerase chain reaction (PCR) analysis according to the manufacturer's protocol. U6 snRNA was used as the endogenous control. For real-time PCR, the cDNA template (2 µl) was mixed with SYBER Green Master Mix (Cat No. 218073) (Qiagen, CA, USA) in a final volume of 25 µl and added to a custom 96-well miScript miRNA PCR array plate (Cat No. MIHS-113Z) (Qiagen, CA, USA), which was enriched with forward and reverse miRNA-specific primers for miR-92a, miR-143 and miR-342. Optical thin-wall 8-cap strips were used to seal the plate. Applied Biosystems 7500 Real Time PCR System (Foster city, CA, USA) was used to perform real-time PCR reactions. The real-time PCR conditions were: 95 C for 15 min, followed by 40 cycles at 94 C for 15 s, 55 C for 30 s and 70 C for 34 s. The number of cycles required for the fluorescent signal to cross the threshold (background noise level) was calculated to determine the cycle threshold (CT) in real-time PCR. Then the CT values of U6 snRNA were subtracted from the CT values of the target miRNAs to determine the ΔCt values of the three miRNAs. ΔCt values are inversely correlated with miRNAs expression levels. Therefore, lower ΔCt values are correlated with increased miRNAs expression. We used the 2^-ΔΔ (Ct)^ method to calculate the relative quantitative levels of individual miRNAs.

**Statistical analysis**

Comparisons of quantitative variables were performed using the nonparametric Mann–Whitney U test when comparing AML group and control group. The P-values less than 0.05 were considered as statistically significant. Statistical comparison among AML subgroups was performed by one-way analysis of variance (Anova). When a significant overall p value (P<0.05) was present, differences between individual means were tested using Fisher's least-significant differences (LSD). Spearman's rank correlation coefficient was also performed to find the correlations between the expression of miRNAs and other variables, e.g. age, gender, and FAB classification. Receiver operator characteristic (ROC) curves were derived and area-under-the curve (AUC) analysis was performed to obtain the best cut-off value of miRNA value for detecting AML patients. All statistical analyses were performed using SPSS 23 software, and graphs were generated using MedCalc Version 16.8.4 (MedCalc statistical software).

**Table 1:** Clinical-pathological parameters of AML and healthy control groups

	**AML group** **n= 65 (%)**	**Healthy group** **n** ***=*** ** 50 (%)**
**Sex **		
Male	38 (58.5%)	28 (56%)
Female	27 (41.5%)	22 (44%)
**Age**		
Mean±SD	55.28 ± 5.35	54.67 ± 5.83
Median	56	55
Range	43─68	42─67
**French-American-British (FAB) classification system**		
M0	2 (3.1%)	NA
M1	20 (30.8%)	
M2	26 (40%)	
M4	9 (13.8 %)	
M5	7 (10.8%)	
M7	1 (1.5%)	

NA: not applicable; M0: acute myeloblastic leukemia, minimally differentiated; M1: acute myeloblastic leukemia, without maturation; M2: acute myeloblastic leukemia, with granulocytic maturation; M4: acute myelomonocytic leukemia; M5: acute monoblastic or monocytic leukemia; M7: acute megakaryoblastic leukemia

**Fig. 1:**
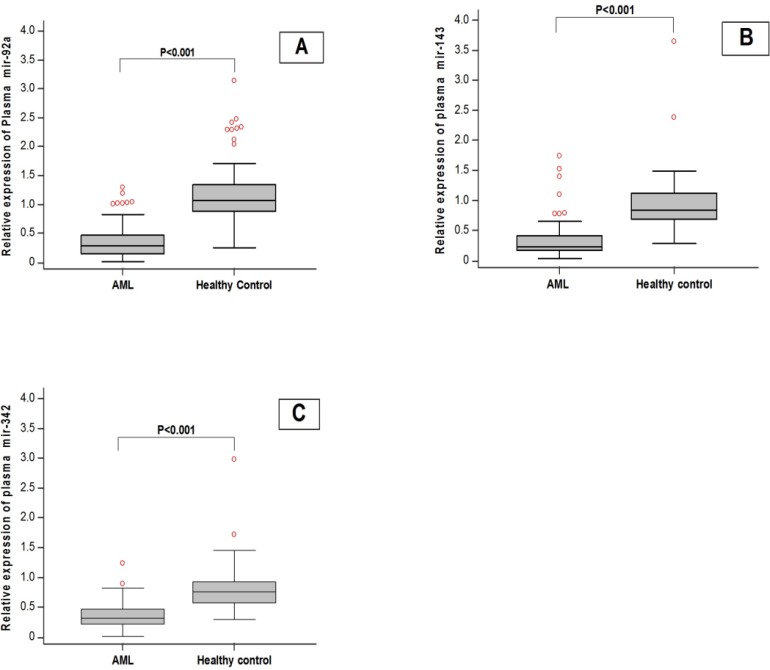
Box plot of plasma levels of miRNAs in healthy normal subjects and acute myeloid leukemia (AML) patients. A: miR-92a; B: miR-143; C: mir-342

## Results

**Expression levels of miRNAs**

**Fig. 2.**
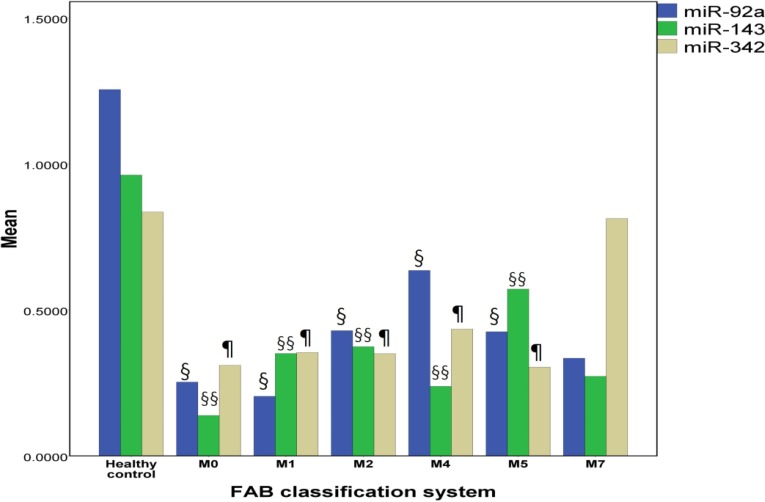
Bar Graph of plasma levels of miR-92a, miR-143, and mir-342 in different AML subtypes. **§**: significant difference in miR-92a expression versus healthy control group; **§§**: significant difference in miR-143 expression versus healthy control group; **¶**: significant difference in miR-342 expression versus healthy control group. (P< 0.05

The data presented in Figure 1 shows that there is a significant decrease in the expression levels of miR-92a, miR-143 and miR-342 in AML patients compared with the control group (P< 0.001). The ratios of the plasma miRNAs in AML patients to healthy controls are displayed in Table 2 with their statistical significance. In the present study, there was no significant difference in the expression of the three miRNAs among AML subtypes (M0, M1, M2, M4, and M5). However, all AML subtypes showed significant decrease in the three miRNAs expression levels compared to healthy normal subjects (Figure 2). As only one AML case with M7 classification was recruited, therefore, group M7 was not included in the post hoc tests because there was a standard deviation of this group.

In addition, no significant correlations were found between the expression levels of any of the three miRNAs and age, gender, and FAB classification in AML patients, except for miR-92a. As shown in Table 3, only miR-92a showed a significant correlation (r=.397, P= 0.01) with FAB classification.

**Table 2:** Circulating plasma microRNAs expression levels in AML patients compared to healthy controls

**microRNA**	**AML/Healthy control ** **Ratio ± SD**	**P value**
**miR-92a**	0.304 ± .147	P<0.0001
**miR-143**	0.369 ± .215	P<0.001
**miR-342**	0.436 ± .084	P<0.001

Data are presented as means ratio ± SD (standard deviation). P < 0.05 was considered as statistically significant

**Diagnostic performance of miR-92a, miR-143 and miR-342 for AML**

**Fig. 3:**
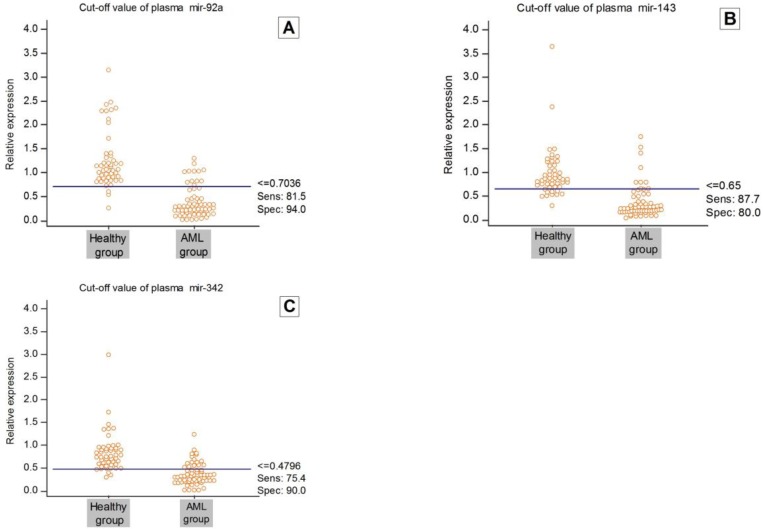
Cut-off values of plasma levels of miR-92a, miR-143, and mir-342. A: miR-92a; B: miR-143; C: mir-342. Cut-off values were determined such that they maximized the sum of sensitivity and specificity. Sens.: sensitivity; Spec.: specificity.

**Fig. 4:**
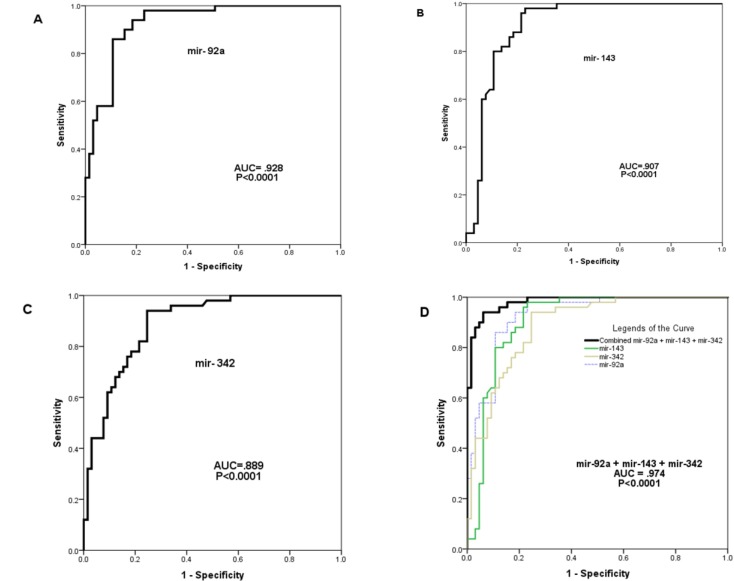
Receiver operating characteristic (ROC) curve analysis. The ROC plots for miR-92a (A), miR-143 (B), miR-342 (C), and combination of three miRNAs (D) were used to differentiate acute myeloid leukemia (AML) patients from normal subjects. AUC: area under the curve.

The diagnostic performance of miR-92a, miR-143 and miR-342 (and a combination of these markers) in differentiating AML patients from healthy volunteers was evaluated by ROC curve analysis. Cut-off points were determined such that they maximized the sum of sensitivity and specificity. The cut-off points for miR-92a, miR-143 and miR-342 were 0.704, 0.65 and 0.479, respectively (Figure 3). The sensitivity and specificity of miR-92a calculated in this study were 81.5% and 94%, respectively, with an AUC value of 0.928 [95% confidence interval (CI), .882 to .974], (Figure 4A). The sensitivity and specificity of miR-143 were 87.7% and 80%, respectively, with an AUC value of 0.907 [95% confidence interval, .849 to .965] (Figure 4B). Finally, the sensitivity and specificity of miR-342 quantitative expression calculated in this study were 75.4% and 90%, respectively, with AUC value of 0.889 [95% confidence interval, .831 to .947] (Figure 4C). The AUC for the combined miR-92a and miR-143 and miR-342 was 0.974 [95% confidence interval, .937 to .988] (Figure 4D).

**Table 3:** Correlation of plasma levels of miRNAs with clinicopathological parameters in AML patients

	**miR-92a**		**miR-143**		**miR-342**	
	**r**	**P**	**r**	**P**	**r**	**P**
**Age**	.047	.711	-.052	.683	.043	.736
**Gender**	-.169	.180	-.072	.571	.180	.152
**FAB classification**	.397	.001**	.194	.121	.130	.301

**r**: Spearman's rank correlation coefficient;

**: Correlation is significant when P< 0.05

## Discussion

In AML patients, early detection and timely intervention can increase survival chances. Consequently, to develop more effective prevention and treatment protocols, there is a need to find new biomarkers of the diagnosis and prognosis of AML. MiRNAs expression profiling has been shown to classify tissue and tumor type accurately in different types of cancers (21, 22). Though analysis of miRNA expression in biopsy samples may be associated with sampling errors and invasiveness, the quantification of plasma miRNA levels is less invasive, simpler and can monitor tumor dynamics. Several reports have confirmed that miRNAs are reliably detectable in plasma and serum. AS a consequence, circulating miRNAs level in the plasma may reflect the expression of miRNAs in tumor tissues (23). Not only miRNAs represent promising biomarkers with potential for detecting AML but miRNAs also predict reliable prognosis and monitor treatment response. In the current study, we focused on expression levels of plasma micoRNAs to determine their usefulness as markers for diagnosing patients with AML (13). We chose the three miRNAs (miR-92a, miR-143 and miR-342) by interpreting the published studies that report the downregulation of these miRNAs in AML cell lines and tumor samples. The study of the expression profile of these miRNAs in plasma presents new non-invasive biomarkers for this type of hematologic malignancy.

In the present study, the expression of miR-92a, miR-143 and miR-342 were downregulated in AML patients’ plasma compared with healthy controls, suggesting the important role of these miRNAs as tumor suppressors in acute leukemia pathogenesis. This study included AML patients with the FAB classification subtypes of M0, M1, M2, M4, M5, and M7, while AML patients with M3 and M6 subtypes were not represented in the study. Though AML has a heterogeneous nature with diverse classifications, there was no significant difference in the expression of the three miRNAs among the AML subgroups (M0, M1, M2, M4, and M5).

The downregulation of plasma miR-92a has been reported in different types of tumors, for example, non-Hodgkin's lymphoma (24) and hepatocellular carcinoma (25). There are few studies that discussed the plasma level of miR-92a in acute leukemia. In a study carried out by Tanaka et.al (14), miR-92a/ miR-638 ratio in 61 AML patients’ plasma was much lower compared to 16 normal control. Tanaka, et al. used miR-638 as internal reference for miR-92a because it is present in human plasma in a stable form. In the same study, miR-92a was highly induced in leukemic cells in both AML and acute lymphoblastic leukemia (ALL) patients. These findings suggested that microRNAs are packed inside exosomes that are secreted from cells to be delivered to other cells and to function in another tissue or organ. It was also proposed that blast cells take in the exosomes containing miR-92a. Accordingly, the level of miR-92a decreases in plasma in AML. Similarly, another study (15) showed decreased plasma levels of miR-92a in 25 newly diagnosed AML patients compared to 25 normal subjects. So, our results agree well with previous studies that reported decreased levels of plasma miR-92a in AML patients.

In an earlier, it was reported that miR-143 is downregulated in the bone marrow cells from 63 acute leukemia patients compared to 15 normal controls. Another finding in this study was the negative correlation between the expression of miR-143 and the expression of DNA methyltransferase 3 alpha (*DNMT3A*) mRNA, which is an identified target gene of miR-143. This study also proposed that miR-143 may play a major role in leukemia-cell proliferation and apoptosis, probably by silencing of *DNMT3A* (16). Batliner et al. (26) also found that neutrophils from AML patients have a significant decrease in the expression of miR-143 and miR-145 compared to neutrophils of healthy donors. This downregulation of miR-143 and miR-145 was attributed to the overexpression of p73, which functions as transcriptional regulator of miR-143/145 during neutrophil differentiation. Several studies discussed the target genes of miR-143 and its potential functions. It was reported that miR-143 inhibits tumor proliferation and promotes apoptosis by targeting BCL-2 in cervical cancer (27). This finding was confirmed by Zhang et al. (28) who identified the proapoptotic function of miR-143 in osteosarcoma pathogenesis by targeting BCL-2. Another potential function of miR-143 was reported by Fang et al. (29) who identified miR-143 as an essential regulator of cancer glycolysis via targeting hexokinase 2 gene (*HK2*). To date, we are the first to report the decreased level of miR-143 in the plasma of AML patients.

Downregulation of miR-342 in 20 AML patients was previously demonstrated by Fayyad-Kazan et al. (17). They showed that plasma miR-342 has a good sensitivity and specificity in diagnosing AML with ROC AUC of 0.8125. In agreement with this previous study, we confirmed the potential role of miR-342 in AML diagnosis. Earlier, it was reported that miR-342-3p acts as a tumor suppressor in non-small cell lung cancer through repression of Ras-related protein Rap-2b (*RAP2B*) (30). Another study reported that miR-342 inhibited tumor development and induced apoptosis in prostate cancer cells by blocking sterol regulatory element-binding protein (SREBP) metabolic pathway (31). In colorectal cancer (CRC) tissues and cell lines, miR-342 functioned as a tumor suppressor gene in CRC development by targeting DNA methyltransferase 1 (*DNMT1*) and aberrant DNA hypermethylation (32).

We employed the ROC curve to analyze the diagnostic value of plasma miR-92a, miR-143 and miR-342 levels in AML patients. subsequently, ROC demonstrated that miR-92a and miR-143 had a high diagnostic value for AML with the AUC of 0.928 and 0.907, respectively. MiR-342 showed a moderate diagnostic value for AML with the AUC of 0.889. More powerful diagnostic values were observed when combining the three miRNAs (miR-92a, miR-143 and miR-342), resulting in an AUC of 0.974.

Although our results are promising, this study has several limitations. First, as the sample size is still small, further validations in large cohorts or in different ethnic groups are recommended. Second, it is uncertain whether this decreased level of plasma miRNAs is very specific for hematological malignancies. Then, additional studies are necessary to compare their plasma levels in different types of cancer to validate the specificity of these miRNAs to AML.

In conclusion, this study shows a reduction in miR-92a, miR-143 and miR-342 levels in plasma of AML patients. The combination of the plasma markers miR-92a, miR-143 and miR-342 represents a very promising screening test for AML diagnosis. Although testing with these markers reached high sensitivity and specificity for AML prediction, there is a need to compare the results with other types of cancer to clarify whether these miRNAs are useful as specific markers for AML.
